# Assessment of pain and postoperative nausea and vomiting and their association in the early postoperative period: an observational study from Palestine

**DOI:** 10.1186/s12893-021-01172-9

**Published:** 2021-04-01

**Authors:** Reem M. Elsaid, Ashraqat S. Namrouti, Ahmad M. Samara, Wael Sadaqa, Sa’ed H. Zyoud

**Affiliations:** 1 Department of Medicine, College of Medicine and Health Sciences, An-Najah National University, 44839 Nablus, Palestine; 2 Department of Anesthesia and Intensive Care, An-Najah National University Hospital, 44839 Nablus, Palestine; 3 Department of Clinical and Community Pharmacy, Department of Pharmacy, College of Medicine and Health Sciences, An-Najah National University, 44839 Nablus, Palestine; 4 Poison Control and Drug Information Center (PCDIC), College of Medicine and Health Sciences, An-Najah National University, 44839 Nablus, Palestine; 5 Clinical Research Center, An-Najah National University Hospital, 44839 Nablus, Palestine

**Keywords:** Postoperative nausea and vomiting, Postoperative pain, Surgical patients

## Abstract

**Background:**

Postoperative nausea and vomiting (PONV) and postoperative pain (POP) are most commonly experienced in the early hours after surgery. Many studies have reported high rates of PONV and POP, and have identified factors that could predict the development of these complications. This study aimed to evaluate the relationship between PONV and POP, and to identify some factors associated with these symptoms.

**Methods:**

This was a prospective, multicentre, observational study performed at An-Najah National University Hospital and Rafidia Governmental Hospital, the major surgical hospitals in northern Palestine, from October 2019 to February 2020. A data collection form, adapted from multiple previous studies, was used to evaluate factors associated with PONV and POP in patients undergoing elective surgery. Patients were interviewed during the first 24 h following surgery. Multiple binary logistic regression was applied to determine factors that were significantly associated with the occurrence of PONV.

**Results:**

Of the 211 patients included, nausea occurred in 43.1%, vomiting in 17.5%, and PONV in 45.5%. Multiple binary logistic regression analysis, using PONV as a dependent variable, showed that only patients with a history of PONV [odds ratio (OR) = 2.28; 95% confidence interval (CI) = 1.03–5.01; *p* = 0.041] and POP (OR = 2.41; 95% CI = 1.17–4.97; *p* = 0.018) were significantly associated with the occurrence of PONV. Most participants (74.4%) reported experiencing pain at some point during the first 24 h following surgery. Additionally, the type and duration of surgery were significantly associated with POP (p-values were 0.002 and 0.006, respectively).

**Conclusions:**

PONV and POP are common complications in our surgical patients. Factors associated with PONV include a prior history of PONV and POP. Patients at risk should be identified, the proper formulation of PONV protocols should be considered, and appropriate management plans should be implemented to improve patients’ outcomes.

**Supplementary Information:**

The online version contains supplementary material available at 10.1186/s12893-021-01172-9.

## Background

Efficacy of healthcare administration, raising the patient’s level of satisfaction, and obtaining optimal outcomes are the major reflection tools for evaluating any health system's overall quality. Multiple postsurgical complications with different severities have been recorded, including postoperative nausea and vomiting (PONV) and perioperative pain [1, 2]. PONV is defined as the experience of nausea, vomiting, or both during the first 24 h following surgery. It is one of the most reported causes of distress and dissatisfaction among surgical patients in the early postoperative hours. It is frequently cited as one of the most unpleasant complications that patients would prefer to avoid, and successful prevention of this complication greatly improves their satisfaction and hastens their resumption of daily activities [3, 4].

PONV affects the decision of when to discharge patients from the recovery room and is associated with a delayed hospital stay, thus increasing ward burden and overall healthcare costs. Although typically self-limiting and rarely fatal, PONV increases the patients’ risk of developing dehydration, serum electrolyte disturbances, aspiration and subsequent pneumonia, and, if severe enough, oesophageal rupture [5].

Recent studies showed that the incidence of PONV continues to be unacceptably high, ranging from 9 to 56% [6–8]. Furthermore, in high-risk patients undergoing surgery without receiving any prophylaxis, the incidence of PONV was reported to be as high as 80% [9, 10]. However, there is still a shortage of studies that discuss the pathophysiology and potential factors influencing the development of PONV, which might contribute to its high incidence rate [5, 11]. Many factors are proposed as risk factors for PONV. These can be categorized into patient-related and surgery-related factors. The Apfel score assesses four factors: sex, smoking status, postoperative opioid use, and prior motion sickness or PONV. Assessment of the patients’ risk of experiencing PONV helps in predicting this problematic postoperative consequence [6].

Another highly reported complication in the postoperative period is pain [12]. Several studies have addressed this complication and helped improve our understanding of its underlying mechanisms. However, postoperative pain (POP) continues to be a prevalent yet unresolved concern [13, 14]. Recognizing factors associated with POP provides the basis for effective pain management and improved outcomes [15]. POP is also associated with other public health and economic concerns [13]. Therefore, effective pain management is also the key to achieving better outcomes on the societal level [16].

In Palestine, there is data on the incidence of PONV, the factors associated with it, and the association between PONV and POP. Evaluating pain and PONV is noteworthy because the key reasons for the failure of discharge after day-case surgery are pain and PONV. Therefore, this study aimed to determine whether PONV and POP were associated with demographic or surgical characteristics among patients recovering from surgery and to evaluate the relationship between PONV and POP. The results of this study provide valuable data that can help in improving surgical outcomes for patients and the healthcare system at large.

## Methods

### Study design and setting

This was a prospective, multicentre, observational study conducted to estimate the incidence of PONV in surgical patients and their associations with different pain indices. The study was conducted at An-Najah National University Hospital, a large tertiary hospital and an important referral centre for patients from both the West Bank and Gaza, and Rafidia Governmental Hospital, a major surgical hospital in the northern West Bank. Both hospitals are in Nablus city in the West Bank, Palestine. Data was collected between October 2019 and February 2020. This study adhered to the STROBE guideline.

### Study area and population of the study

Our target population was surgical patients in Palestine. Participants were enrolled in this study based on the specified criteria for inclusion and exclusion.

### Sample size and sampling technique

A total of 211 patients undergoing elective surgery under general anaesthesia at An-Najah National University Hospital and Rafidia Surgical Governmental Hospital were selected using a convenient sampling procedure.

### Inclusion and exclusion criteria

Patients aged above 18 years old of both sexes who were scheduled for elective surgery under general anaesthesia and agreed to participate in our study were eligible to participate. Our exclusion criteria included admission to the intensive care unit (ICU), requiring the insertion of a nasogastric tube after surgery, having cognitive impairment, or receiving steroids or antiemetic medications in the last 24 h before surgery.

### Data collection instruments

We conducted data collection form-based interviews with the participants during the first 24 h following their surgery. The data collection form consisted of four sections [17–19] (Additional file 1: Data collection form). The first section contained questions inquiring about the participant’s demographic (age and sex) and clinical (BMI, smoking, history of motion sickness and history of PONV) characteristics. The second section inquired about their current operation, including the type of surgery, duration of surgery, postoperative opioid use and POP.

The third section assessed postoperative nausea (defined as pronounced stomach discomfort with the sensation of an urge to vomit) using a visual analogue scale (VAS) that was approved for assessing the intensity of nausea in the postoperative period [17]. Participants rated the overall intensity of nausea they felt during the first 24 h of the postoperative period on a 100-mm VAS, with 0 meaning no nausea at all and 100 meaning severe nausea. In this section, we also collected data on postoperative vomiting by asking about the number of vomiting episodes experienced in the 24 h following surgery. PONV, defined as suffering from postoperative nausea, vomiting, or both, was also assessed in this section.

The last section contained items that assessed POP severity during the first 24 h following surgery using a numerical rating scale [18]. Participants reported their response as a number from 0 to 10, with 0 meaning no pain at all, 1–3 meaning mild pain, 4–6 meaning moderate pain and 7–10 meaning severe pain. We recorded the severity of the average overall pain, as well as the severity of pain on movement (defined as pain experienced with any active movement, including getting out of bed, sitting, turning to the side, defecating, taking deep breaths and coughing forcefully) and pain at rest (defined as pain occurring while in bed with no active movements).

Data on anaesthetic drugs and analgesic administration was collected from the intraoperative anaesthetic charts and the inpatients electronic recording system after obtaining consent to use this data from both the hospital and the participants.

The data collection form was pilot-tested on 20 patients at An-Najah National University Hospital.

### Ethical approval

The study was reviewed and approved by the *Institutional Review Board (IRB) of An-Najah National University*. Participants had the right to choose whether they would like to be included in the study or not. Verbal consent was obtained, and this was followed by providing the participant with written informed consent to read and sign voluntarily.

### Statistical analysis

All statistical analysis was conducted using version 21.0 of Statistical Package for Social Sciences (SPSS) (IBM Corp., Armonk, NY, USA). Variables were presented as frequencies and percentages and/or medians and interquartile ranges, as appropriate. Pearson’s chi-square test was used for correlational analysis between variables, and p-values were reported. For PONV assessment, we divided our participants into two groups: those who had PONV and those who did not have PONV. All univariate variables significant at *p* < 0.05 were entered into a multiple binary logistic regression model to determine factors that were significantly associated with the occurrence of PONV. By estimating the odds ratio (OR) at a 95% confidence interval (CI), the association between the occurrence of PONV and each independent variable was measured. For pain assessment, patients were categorized into two groups: those who had POP and those who did not have POP. A p-value less than 0.05 was considered statistically significant.

## Results

### Demographic and clinical characteristics and their association with PONV

**Fig. 1:**
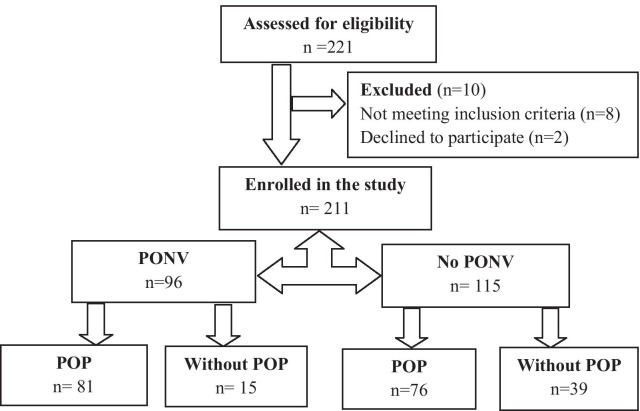
Flow diagram of the study patients. *PONV* postoperative nausea and vomiting, *POP* postoperative pain

Out of the 221 subjects approached, 211 were included in this study, accounting for a response rate of 95.5%. The demographic and clinical characteristics of the participants are presented in Table 1. The median age of the participants was 33 years, with an interquartile range (IQR) of 24–48. The male-to-female ratio was roughly balanced (55.5% and 44.5%, respectively). Almost half (46.4%) of the participants were overweight (BMI in the 25–29.9 range) and the majority had no history of motion sickness (87.7%) or PONV (82.5%). Figure 1 shows a flow diagram of the study patients. Using the Apfel simplified score, participants can be categorized depending on the number of risk factors they have, into 0, 1, 2, 3 or 4 risk factors, as shown in Table 2. Most of the participants had only 1 or 2 risk factors (30.3% and 31.3%, respectively).Of the 211 participants, 43.1% reported experiencing nausea, whereas only 17.5% reported having vomited. The overall incidence of PONV in our sample was 45.5% (Table 2).

**Table 1:** Demographic and clinical characteristics and their relationship with PONV

Characteristic	Total; N = 211 (%)	PONV; N = 96 (%)	No PONV; N = 115 (%)	P-value*
Age (year)				
18–39	135 (64)	59 (61.5)	76 (66.1)	0.658
40–64	66 (31.3)	33 (34.4)	33 (28.7)	
65–79	10 (4.7)	4 (4.2)	6 (5.2)	
Sex				
Female	94 (44.5)	48 (50.0)	46 (40)	0.146
Male	117 (55.5)	48 (50.0)	69 (60)	
BMI				
Underweight	4 (1.9)	3 (3.1)	1 (0.9)	0.208
Healthy weight	57 (27.0)	21 (21.9)	36 (31.3)	
Overweight	98 (46.4)	50 (52.1)	48 (41.7)	
Obese	52 (24.6)	22 (22.9)	30 (26.1)	
Smoking	93 (44.1)	40 (41.7)	53 (46.1)	0.520
History of motion sickness	26 (12.3)	10 (10.4)	16 (13.9)	0.442
History of PONV	37 (17.5)	24 (25.0)	13 (11.3)	**0.009**

*PONV* postoperative nausea and vomiting, *BMI* body mass index

*Significant p-values are in bold

**Table 2:** Apfel score risk factors

Apfel score	Frequency	PONV; N = 96	No PONV; N = 115	P value
Apfel score = 0	46 (21.8)	17 (17.7)	29 (25.2)	0.240
Apfel score = 1	64 (30.3)	28 (29.2)	36 (31.3)	
Apfel score = 2	66 (31.3)	31 (32.3)	35 (30.4)	
Apfel score = 3	29 (13.7)	15 (15.6)	14 (12.2)	
Apfel score = 4	6 (2.8)	5 (5.2)	1 (0.9)	
Overall	211 (100)	96 (100)	115 (100)	

Reported PONV was significantly correlated with a history of PONV among the participants (*p* = 0.009). No other significant association was observed between reported PONV and demographic or clinical characteristics (Table 1).

### Operation-related factors and their association with PONV

The most frequent types of surgery that participants underwent were general surgery/laparotomy (30.8%), laparoscopic surgery (21.3%) and orthopaedic surgery (12.3%). Table 3 presents the percentage of participants based on the type of surgery they underwent and shows the association of PONV with operation-related factors among the participants. The type of surgery showed a significant association with PONV (*p* = 0.010). Additionally, we found a significant association between the occurrence of PONV and experiencing POP (*p* = 0.002).

**Table 3:** Operation-related factors and their association with PONV

Characteristic	Total; N = 211 (%)	PONV; N = 96 (%)	No PONV; N = 115 (%)	P-value*
Type of surgery				
General	65 (30.8)	30 (31.3)	35 (30.4)	**0.010**
Laparoscopic	45 (21.3)	26 (27.1)	19 (16.5)	
Orthopedic	26 (12.3)	15 (15.6)	11 (9.6)	
Otorhinolaryngology	35 (16.6)	11 (11.5)	24 (20.9)	
Urosurgery	21 (10.0)	5 (5.2)	16 (13.9)	
Neurosurgery	11 (5.2)	8 (8.3)	3 (2.6)	
Vascular surgery	4 (1.9)	1 (1.0)	3 (2.6)	
Gynecological surgery	4 (1.9)	0 (0.0)	4 (3.5)	
Duration of surgery				
< 60 min	114 (68.2)	63 (65.6)	81 (70.4)	0.455
≥ 60 min	67 (31.8)	33 (34.4)	34 (29.6)	
Post-operative opioid	50 (23.7)	27 (28.1)	23 (20.0)	0.167
Post-operative pain	157 (74.4)	81 (84.4)	76 (66.1)	**0.002**

*PONV* postoperative nausea and vomiting, *BMI* body mass index

*Significant p-values are in bold

### Occurrence and severity of POP and its association with PONV

Most participants (74.4%) reported experiencing some degree of POP (Table 3), with 14.2% experiencing severe pain at rest and 29.4% experiencing severe pain with movement. Data on the occurrence and severity of POP, in general and per set, is presented in Table 4. Of all participants with POP, 46.9% underwent short operations (surgery duration of < 60 min). There were also significant associations between PONV and pain severity in general, at rest, and with movement (p-values were < 0.001, < 0.001, and 0.010, respectively), as shown in Table 4. Both the type and duration of surgery were significantly correlated with the occurrence of POP (p-values were 0.002 and 0.006, respectively). Table 5 shows the association of POP with operation-related factors among the participants.

**Table 4:** The occurrence and severity of postoperative pain and their association with PONV

Characteristic	Frequency (%)	PONV; N = 96 (%)	No PONV; N = 115 (%)	P-value*
Pain severity				
None	54 (25.6)	15 (27.8)	39 (72.2)	**< 0.001**
Mild	56 (26.5)	22 (39.3)	34 (60.7)	
Moderate	64 (30.3)	31 (48.4)	33 (51.6)	
Severe	37 (17.5)	28 (75.7)	9 (24.3)	
Pain at rest				
None	54 (25.6)	15 (27.8)	39 (72.7)	**< 0.001**
Mild	72 (34.1)	34 (47.2)	38 (52.8)	
Moderate	55 (26.1)	23 (41.8)	32 (58.2)	
Severe	30 (14.2)	24 (80.0)	6 (20.0)	
Pain with movement				
None	54 (25.6)	15 (27.8)	39 (72.2)	**0.010**
Mild	56 (26.5)	25 (44.6)	31 (55.4)	
Moderate	39 (18.5)	20 (51.3)	19 (48.7)	
Severe	62 (29.4)	36 (58.1)	26 (41.9)	

*PONV* postoperative nausea and vomiting

*Significant p-values are in bold

**Table 5:** Operation-related factors and their association with pain occurrence

Operation-related factors	With post-operative pain; N = 157 (%)	Without post-operative pain; N = 54 (%)	P value*
Type of surgery			
General surgery/Laparotomy	51 (32.5)	14(25.9)	**0.002**
Laparoscopic	38 (24.2)	7 (13.0)	
Orthopedic	22 (14.0)	4 (7.4)	
Otorhinolaryngology	24 (15.3)	11 (20.4)	
Urosurgery	9 (5.7)	12 (22.2)	
Neurosurgery	8 (5.1)	3 (5.6)	
Vascular	1 (0.5)	3 (5.6)	
Gynecological	4 (1.9)	0 (0.0)	
Duration of surgery			
< 60 min	99 (63.1)	45 (83.3)	**0.006**
≥ 60 min	58 (36.9)	9 (16.7)	

*Significant p-values are in bold

### Use of drugs and their association with both PONV and pain

Despite the diversity of operations that our participants underwent, almost all participants received the same anaesthetic drugs. Propofol, fentanyl, midazolam and atracurium were a standardized regimen. The rates of PONV in association with these drugs were 100%, 99%, 38.5% and 53.1%, respectively. Table 6 shows the percentage of usage of these drugs, as well as the frequency of experiencing PONV and POP. Most (69.4%) of the patients who reported experiencing pain in the postoperative period were given opioids. Among those, 28.1% experienced PONV, whereas 71.9% of the 161 patients (76.3% of all participants) who were managed with non-opioid analgesics (mainly acetaminophen) had PONV.

**Table 6:** Association between PONV, pain and the drugs used

Medications	PONV; N = 96 (%)	No PONV; N = 115 (%)	P value*	Pain; N = 157	No pain; N = 54	P value*
Propofol	96 (100)	113 (98.3)	0.296	155 (98.7)	54 (100)	0.553
Fentanyl	95 (99)	113 (98.3)	0.568	156 (99.4)	52 (96.3)	0.162
Midazolam	37 (38.5)	42 (36.5)	0.436	60 (38.2)	19 (35.2)	0.410
Atracurium	51 (53.1)	58 (50.4)	0.401	83 (52.9)	26 (48.1)	0.330
Opioid	27 (28.1)	23 (20)	0.111	109 (69.4)	52 (96.3)	**0.000**

*Significant p-values are in bold

### Multiple logistic regression analysis

Variables with a *p*-value < 0.05, including the history of PONV, POP and type of surgery, were entered in a multiple binary logistic regression model. Some of the above associations did not exist after controlling other variables. In multiple binary logistic regression analysis using PONV as a dependent variable, only patients with a history of PONV (OR = 2.28; 95% CI = 1.03–5.01; *p* = 0.041) and POP (OR = 2.41; 95% CI = 1.17–4.97; *p* = 0.018) were significantly associated with the occurrence of PONV (Table 7). All included variables in the multiple binary logistic regression had a significant *p*-value in the univariate analysis between PONV and non‐PONV groups. The model was significant, with a Chi‐square of 28.67, DF = 8; *p* < 0.001.

**Table 7:** Independent factors associated with PONV using multiple logistic regression analysis (enter method)

Variable	B	S.E	Wald	P value*	Odds ratio with 95% CI
History of PONV					
No					Ref
Yes	0.82	0.40	4.18	0.041	2.28 (1.03–5.01)
Post-operative pain					
No					Ref
Yes	0.88	0.37	5.64	0.018	2.41 (1.17–4.97)
Type of surgery					
General					Ref
Laparoscopic	0.52	0.40	1.64	0.200	1.68 (0.76–3.69)
Orthopedic	0.40	0.48	0.77	0.401	1.50 (0.58–3.85)
Otorhinolaryngology	− 0.48	0.45	1.11	0.292	0.62 (0.26–1.51)
Urosurgery	− 0.59	0.59	0.97	0.324	0.56 (0.17–1.78)
Neurosurgery	1.16	0.75	2.40	0.122	3.17 (0.74–13.67)
Vascular surgery	− 1.52	1.11	1.86	0.172	0.22 (0.26–1.94)

*PONV* postoperative nausea and vomiting, *CI* confidence interval, *B* the coefficient of the predictor variables

*Significant p-values are in bold

## Discussion

In the current study, we assessed the incidence of PONV and POP, their association, and other factors that could predict their occurrence in surgical patients. This was the first study of its kind in Palestine. We found that PONV and POP are common complications in the first 24 h following surgery. We also reported a significant association between these two complications, as well as their association with other factors. PONV was associated with previous PONV and surgery type, whereas POP was associated with surgery duration.

PONV and POP significantly affect morbidity and patient satisfaction in the immediate postoperative period. The overall incidence of PONV, as described in our study, is comparable to the findings of a 2012 systematic review that included 22 studies [6].

Factors that showed a statistically significant association with PONV in the 2012 review included previous PONV, which was an association that we also found to be significant in our sample. It is postulated that a history of PONV points to the presence of an underlying susceptibility to PONV [6]. This finding was also in agreement with the findings of other studies that were conducted in Ethiopia [20], Korea [21] and Uganda [22].

A possible explanation for this finding was suggested by a 2011 study that used pooled DNA samples to detect genetic markers that would potentially influence the possibility of experiencing PONV [23]. In that study, subjects with previous PONV were more likely to have first-degree relatives with a history of PONV [23].

The presence of motion sickness history, on the other hand, did not have a significant effect on experiencing PONV in our study, similar to the findings of a study that examined 174 patients undergoing minor orthopaedic surgery [24]. Another more recent study reported similar findings on the relationship between a history of motion sickness and PONV [22].

Our results also showed that the type of operative procedure and the incidence of PONV were both significantly associated with experiencing PONV. Similar findings were also reported by a study conducted on 17,638 surgical patients to detect predictors of PONV [25] and by another study that was designed to examine the influence of the type of surgery on the occurrence of PONV [26].

Neither age nor sex was a significant predictor of the occurrence of PONV in this study, similar to the results of a study conducted in Ghana [27]. Another similar prospective, interview-based survey conducted on 1,107 patients between 4 and 86 years old did not find any effect of age on the occurrence of nausea [28]. Furthermore, one study reported an equal incidence of PONV in both males and females undergoing extra-abdominal operations [29]. Although the female sex was found to be a strong risk factor for PONV in some studies, this effect might or might not be mediated by other factors and requires more investigation. For example, it has been proposed that serum progesterone levels, the day of the menstrual cycle, and menopause might have a confounding effect [27]. A retrospective review that examined the influence of the menstrual cycle on PONV found that the incidence was at its highest on day five of the cycle and that there was no nausea and vomiting on days 18–20 [30]. Ethnicity may also have a major role in explaining these results. A prospective study that was conducted to compare the incidence of PONV in different ethnicities concluded that ethnicity could be an independent risk factor for developing PONV [31].

Although non-smoking status and postoperative opioid use were among the predictors of PONV in Apfel’s predictive model [19], no such associations were significant in the current study, similar to other studies from Ethiopia [20] and Uganda [22]. Furthermore, a study that assessed PONV in patients undergoing arthroscopic surgery concluded that short-acting opioids did not have a significant effect on patients’ risk of developing PONV [32]. These findings suggest that the effect of smoking and opioid use on PONV might vary, depending on other patient- and surgery-related factors, and should be investigated further. For example, previous studies have found that the type of surgery might affect the incidence of vomiting upon changing the route of administration in patients receiving morphine, and that vomiting might occur more frequently in patients undergoing abdominal operations compared to hip replacement procedures and in patients receiving epidural opioids compared to intravenous patient-controlled analgesia [33, 34]. Another study reported that intravenous injection of morphine or ketobemidone relieved 80% of nausea episodes [34]. It was also reported that opioid use could rarely lead to postoperative nausea in immobile patients [35].

A high number of patients in this study reported moderate to severe pain during the first 24 h following surgery, similar to findings reported by other studies [8, 11, 36]. Additionally, POP was significantly associated with the development of PONV, which is also similar to reports in the literature [34, 35]. For example, a prospective study that aimed to examine predictors of PONV reported a higher frequency of PONV among patients experiencing excessive POP [25]. A significant association between reported pain and PONV was also seen in several other studies [26, 37, 38]. A study conducted in Uganda found that surgical patients who reported feeling pain were twice as likely to experience PONV than patients who did not [22]. It was also noted that nausea was frequently accompanied by pain in the first few hours after surgery, and that relieving the pain led to a decrease in nausea [25, 35]. Similarly, a study conducted in Norway found that managing pain reduced nausea symptoms [35]. These findings are in agreement with findings from previous studies that concluded that the use of intravenous acetaminophen postoperatively might reduce PONV incidence and the need to give antiemetic medications [39, 40].

Several methods have been used in the recovery room to minimize POP. Opioids continue to be a popular means, despite their widely recognized adverse effects. Recently, non-opioid analgesics and adjuncts, such as dexamethasone, have been used as opioid-sparing alternatives [41]. Recent guidelines recommend using second-generation 5-hydroxytryptamine 3 (5-HT3) receptor antagonists, dopamine antagonists and neurokinin 1 (NK1) receptor antagonists as prophylaxis and treatment options for nausea and/or vomiting in the postoperative setting [42].

In summary, the current study found a high PONV incidence among surgical patients and identified factors associated with PONV, including previous PONV, type of surgery and POP. Additionally, we found a high incidence of POP, necessitating the implementation of appropriate pain management protocols during the postoperative period.

### Strengths and limitations

The current study had some limitations. Participants were interviewed within 24 h following their surgery, which may have limited our findings to symptoms occurring inside this time window. Moreover, our study did not include PONV and POP prophylaxis and management protocols followed by the hospitals studied. Furthermore, the high variability of the population selected (e.g., pain, opioid consumption, type of surgery, Apfel risk factors, etc.) might interfere with the statistical analysis results. The final limitation is the lack of information regarding pain management methods, including whether the subjects received regional, local or parenteral analgesia. Thus, we are unable to assess if the methods of analgesia affect the occurrence of POP. On the other hand, this study had many strengths. This was the first study to assess PONV, POP, and their association in Palestine. We also sampled our participants from two of the largest surgical hospitals in the country, which may have improved the generalizability of our findings.

## Conclusions

In conclusion, our findings suggest that PONV and POP are common complications in our surgical patients. Risk factors of PONV include prior history of PONV and POP. Patients at risk should be identified, the proper formulation of PONV protocols should be considered, and appropriate POP management should be highlighted and reinforced to improve patients’ outcomes during the postoperative period.

## Supplementary Information

**Additional file 1: **Data collection form. This is the final version of the English version that was used to obtain data to evaluate the relationship between postoperative nausea and vomiting and postoperative pain, and to identify some factors associated with these symptoms.

## Data Availability

The datasets used and/or analysed during the current study are available from the corresponding author on reasonable request.

## Abbreviations

BMI: Body mass index
CI: Confidence interval
ICU: Intensive care unit
IQR: Interquartile range
IRB: Institutional review board
OR: Odds ratio
PONV: Postoperative nausea and vomiting
POP: Postoperative pain
VAS: Visual analogue scale
