# Supplementary material for: Assessment of pain and postoperative nausea and vomiting and their association in the early postoperative period: an observational study from Palestine
Source: BMC Surg. 2021 Apr 1;21:177. doi: 10.1186/s12893-021-01172-9 (PMC8017875; doi:10.1186/s12893-021-01172-9)
Supplement: Supplementary file 1 — Additional file 1: Data collection form. This is the final version of the English version that was used to obtain data to evaluate the relationship between postoperative nausea and vomiting and postoperative pain, and to identify some factors associated with these symptoms. [file 12893_2021_1172_MOESM1_ESM.doc]

**Additional file 1.** **Data collection form. This is the final version of the** **English version that was used to obtain data which will help to evaluate the relationship between Postoperative nausea and vomiting and postoperative pain, and to identify some factors associated with these symptoms**

**English version**

**Data collection form**

**Section 1**

................................ 1-Age

2-Sex ❑Male ❑Female

3-Height...................

4-Weight..................

5- Residency: ❑city ❑Palestinian refugee camp ❑village

6- Education level: ❑no formal education ❑ primary ❑secondary ❑Graduated

7- Marital status ❑single, widow, divorced ❑ married

8- Occupation ❑Employed ❑Unemployed

9-Are you a smoker? ❑ Yes ❑No

10-have you undergone previous surgeries? ❑ Yes ❑No

11-have you ever experienced PONV before?❑ Yes ❑No

12-have you ever experienced motion sickness before? ❑ Yes ❑No

13-Do you have any diseases?

❑Diabetes mellitus ❑Hypertension ❑Coronary artery disease

❑Stroke ❑Heart Failure ❑Lung disease

Others................................................

14-What are your regular medications?

❑NSAIDS ❑PPI ❑Steroids

❑ Insulin ❑Aspirin ❑Paracetamol

❑anti HTN,………………………………………………………….

❑oral anti-diabetic agents,…………………………………................

Others…………………………………………

**Section 2 (Intraoperative)**

15-type of surgery?

❑ General ❑Orthopaedic ❑ENT ❑Vascular

❑Obstetrics &gynaecology ❑Urology ❑Cardiac ❑Others…………….

16- Surgery technique? ❑Open ❑laparoscopic

17-Anesthitic drugs used………………………………………………………….....

18-Duration of anaesthesia? ❑≤60min ❑>60min

19-Postoperative antiemetic given? ❑ Yes ❑No

20-Perioperative analgesia given?❑ Preoperative ❑Intraoperative ❑Postoperative

21-Opioids given? ❑ Yes ❑No

22- Intraoperative vital signs:

|  | Heart rate | Blood pressure | Temperature | Oxygen saturation |
| --- | --- | --- | --- | --- |
| At induction |  |  |  |  |
| At 15 min |  |  |  |  |
| At 30 min |  |  |  |  |
| At 60min |  |  |  |  |
| >60min |  |  |  |  |
| At recovery room |  |  |  |  |

**Section 3 ( 24hr postoperative)**

23- Have you experienced nausea since your operation? ❑ Yes ❑No

24- If yes, nausea severity rating: ❑ Mild ❑ Moderate ❑ Severe


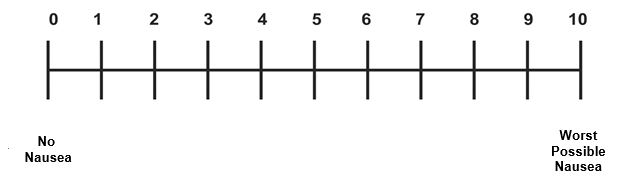


25- Have you experienced vomiting since your operation?

❑ Yes ❑No

26- If yes, number of vomits:

❑ 1-2 ❑ 3-4 ❑ 5-10 ❑ >10

27- Have you experienced pain since your operation?

❑ Yes ❑No

28- If yes, average pain over postoperative 24hr rating:

❑ Mild ❑ Moderate ❑ Severe

29- How is your pain at rest?

❑ Mild ❑ Moderate ❑ Severe

30- How is your pain at movement?

❑ Mild ❑ Moderate ❑ Severe

31- Post operative vital signs?

|  | Heart rate | Blood pressure | Temperature | Oxygen saturation |
| --- | --- | --- | --- | --- |
| At admission to ward |  |  |  |  |
| 4-6 hours |  |  |  |  |
| 24 hours |  |  |  |  |
